# A Preparation of Magnesium

**Published:** 1885-02

**Authors:** 


### A Preparation of Magnesium.

Aprocess, patented by Gratzel, for the separation of alkaline metals by electrolysis, has been very successful in the reduction of magnesium. In Berlin there has recently been exhibited, as a product of this process, a ball of pure magnesium of about five inches diameter. It was exceedingly brilliant, closely resembling silver, and had lost nothing of its luster since its separation. This preservation from corrosion is a sign of the high degree of purity of the metal, and forms a striking contrast to the magnesium hitherto obtained, which was always more or less alloyed with potassium, and consequently easily oxidized, especially in a damp atmosphere. The purer magnesium is considered to be destined to increasing maratime use, because the rays of the magnesium light appear to have a greater penetrative power in fogs and mists than the electric arc.

